# Rod-derived Cone Viability Factor-2 is a novel bifunctional-thioredoxin-like protein with therapeutic potential

**DOI:** 10.1186/1471-2199-8-74

**Published:** 2007-08-31

**Authors:** Frédéric Chalmel, Thierry Léveillard, Céline Jaillard, Aurélie Lardenois, Naomi Berdugo, Emmanuelle Morel, Patrice Koehl, George Lambrou, Arne Holmgren, José A Sahel, Olivier Poch

**Affiliations:** 1 Divisions of Bioinformatics and Biochemistry, Swiss Institute of Bioinformatics, University of Basel, CH-4056 Basel, Switzerland; 2 Laboratoire de Physiopathologie Cellulaire et Moléculaire de la Rétine, Inserm U592, Université Pierre et Marie Curie, 75571 Paris, France; 3 Laboratoire de Biologie et Génomique Structurales, Institut de Génétique et de Biologie Moléculaire et Cellulaire, CNRS/INSERM/ULP, BP 163, 67404 Illkirch cedex, France; 4 Department of Computer Science, Genome Center, University of California, Davis, CA 95616, USA; 5 Novartis Institutes for Biomedical Research, Basel 4002, Switzerland; 6 Medical Nobel Institute for Biochemistry, Department of Medical Biochemistry and Biophysics, Karolinska Institutet, Stockholm, Sweden; 7 Institute of Ophthalmology, University College of London, UK; 8 Fovea-Pharmaceuticals – 12 rue Jean Antoine Le Baif – 75013 Paris

## Abstract

**Background:**

Cone degeneration is the hallmark of the inherited retinal disease retinitis pigmentosa. We have previously identified a trophic factor "Rod-derived Cone Viability Factor (RdCVF) that is secreted by rods and promote cone viability in a mouse model of the disease.

**Results:**

Here we report the bioinformatic identification and the experimental analysis of RdCVF2, a second trophic factor belonging to the Rod-derived Cone Viability Factor family. The mouse RdCVF gene is known to be bifunctional, encoding both a long thioredoxin-like isoform (RdCVF-L) and a short isoform with trophic cone photoreceptor viability activity (RdCVF-S). RdCVF2 shares many similarities with RdCVF in terms of gene structure, expression in a rod-dependent manner and protein 3D structure. Furthermore, like RdCVF, the RdCVF2 short isoform exhibits cone rescue activity that is independent of its putative thiol-oxydoreductase activity.

**Conclusion:**

Taken together, these findings define a new family of bifunctional genes which are: expressed in vertebrate retina, encode trophic cone viability factors, and have major therapeutic potential for human retinal neurodegenerative diseases such as *retinitis pigmentosa*.

## Background

*Retinitis pigmentosa *(RP) is a genetically heterogeneous retinal degeneration characterized by the sequential degeneration of rod and cone photoreceptors. The first clinical signs are night blindness and narrowing of the peripheral field of vision which progressively worsens to become "tunnel-like". Eventually, the central vision is reduced to complete blindness in most cases. At a cellular level, the retinal rod photoreceptors involved in night and side visions slowly degenerate. Subsequently, the cone photoreceptors responsible for both color and high-contrast vision, visual acuity, detail perception and normal light vision are similarly affected. To date, no treatment is available.

This apoptotic degeneration is genetically associated with many mutated loci that encode proteins predominant expressed in retinal rod photoreceptor neurons. The cone loss proposed a paradox since, in a significant proportion of RP patients, the mutated gene is not expressed in these cells. As cones are responsible for the most crucial visual functions, the mechanisms that trigger their degeneration are major therapeutic targets. The retinal degeneration 1 (*rd1*) mouse is the most studied animal model for the human disease. It carries a recessive mutation in the rod-specific cGMP phosphodiesterase beta subunit gene leading to rod photoreceptor death through apoptosis [1,2] followed by cone death presumably through lack of trophic support [3]. We used expression cloning to identify a trophic factor secreted by rods that promotes cone viability in the *rd1 *mouse; RdCVF, for Rod-derived Cone Viability Factor [4]. In the model proposed, rod degeneration results in a decrease of RdCVF expression, which subsequently leads to cone degeneration due to a lack of trophic support [5].

The RdCVF gene, also called thioredoxin-like 6 (Txnl6), encodes the Q8VC33 UniProt [6] protein, which has limited similarity to the thioredoxin superfamily [4]. Thioredoxins (TXN) are usually small proteins which can be involved with pleiotropic activities such as redox control, regulation of apoptosis and cytokine activity [7-9]. The TXN conserved active site contains two distinct cysteines (CXXC) that contribute to a thiol-oxydoreductase activity [9,10] catalyzes the reduction of disulfide bonds in multiple substrate proteins [11,12]. The RdCVF gene encodes two products via alternative splicing: a full length protein and a C-terminal post-transcriptionally truncated protein sharing similarities with TRX80. This latter form of human thioredoxin-1 (Txn) [13-15] has no thiol-reductase activity but is involved in controlling growth of peripheral mononuclear blood cells [13,16]. Similar to Txn, RdCVF looks like a bifunctional gene because it encodes both a long form (RdCVF-L, 217 aa, Q8VC33) having a putative thiol-oxydoreductase activity [17,18] and a short form (RdCVF-S, 109 aa, Q91W38) with trophic activity for cones but no redox activity.

In this paper we report genomic investigations that revealed RdCVF2 as a gene paralogous to RdCVF. Like RdCVF, RdCVF2 is spliced into two alternative mRNAs translated into a long (156 aa, Q9D531) and a short (101 aa, Q91WB0) thioredoxin-like proteins called RdCVF2-L and RdCVF2-S respectively. We explored orthology in available vertebrate genomes and analyzed homology with the thioredoxin superfamily. We also investigated the cone trophic factor activity of RdCVF2 and find it to be similar to that of RdCVF.

## Results

### Identification of RdCVF2, a gene paralogous to RdCVF

**Figure 1:**
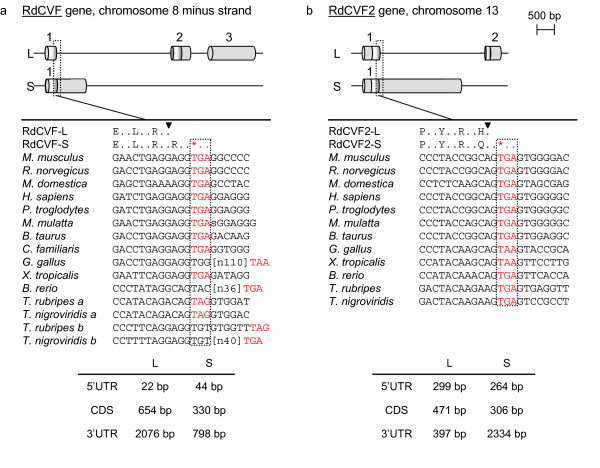
**RdCVF and RdCVF2 gene structure conservation**. At top, panels a and b are the gene structures for RdCVF and RdCVF2 genes. The RdCVF-L mRNA (NM_145598, mouse chromosome 8, minus strand, from 70'033'763 to 70'027'717) is composed of three exons (1–3) of 348, 687 and 1751 bp. The RdCVF-S mRNA (BC017153, from 70'033'785 to 70'032'615) is composed of one exon (1172 bp). The RdCVF2-L mRNA (AK015847, mouse chromosome 13, plus strand, from 50'202'630 to 50'206'797) is composed of two exons (1–2) of 603 and 564 bp. The RdCVF2-S mRNA (BC016199, from 50'202'667 to 50'205'571) is composed of one exon (2904 bp). Coding and non-coding regions are depicted in dark grey) and light grey respectively. At middle, panels a and b, the genomic region surrounding the stop codon at the end of the first coding exon and the corresponding orthologous sequences in 12 other vertebrate genomes are aligned. The black triangles indicate the end of the first RdCVF(2)-L coding exon. Conserved stop codons are colored in red. At bottom, panels a and b, lengths of the coding (CDS) and terminal untranslated regions (UTR) are given.

The mouse RdCVF gene is located on chromosome 8 and contains three exons (Figure 1, panel a). The RdCVF-S splice variant is composed of a single exon in which the coding sequence is the same as the first exon of the long form extended by one codon followed by a stop codon (TGA) and finally a 3' untranslated region (UTR). Consequently, the last 109 amino acids, called the "cap" (see below) of RdCVF-L are missing in RdCVF-S. We identified a paralogous gene on chromosome 13 that we call RdCVF2 (panel b). Both sequence and gene structure are highly similar between the two. Indeed RdCVF2 also encodes both a thioredoxin-like protein (156 aa, Q9D531) and a shorter form (101 aa, Q91WB0) called RdCVF2-L and RdCVF2-S respectively. The degree of homology between RdCVF and RdCVF2 is 58.0% for the long isoforms and 53.5% for the short isoforms.

### Conservation of RdCVF and RdCVF2 gene structure during evolution

Cone viability is related to the production of the RdCVF-S form and, by extension, to the presence of the stop codon at the end of the first exon required to obtain that isoform. To evaluate conservation of that stop codon further, we mapped the RdCVF and RdCVF2 genes on vertebrate genomes available on the UCSC genome browser web site [19] [see Additional file 1). Both loci were found in 13 vertebrates. All these organisms exhibited both genes except *Takifugu rubripes *and *Tetraodon nigroviridis*, in which RdCVF was duplicated at the same chromosomal location (RdCVF a and b) with an additional intron inserted into the first coding exon of this loci. It is noteworthy that the stop codon at the end of the first exon is strictly conserved in the vast majority (Figure 1, panel a and b). This observation implies the possible existence of RdCVFs short isoforms in most vertebrates, excepting *Gallus gallus *and *Brachydanio rerio *RdCVF; *Tetraodon nigroviridis *and *Takifugu rubripes *RdCVFb.

### Analysis of RdCVF and RdCVF2 protein sequences

**Figure 2:**
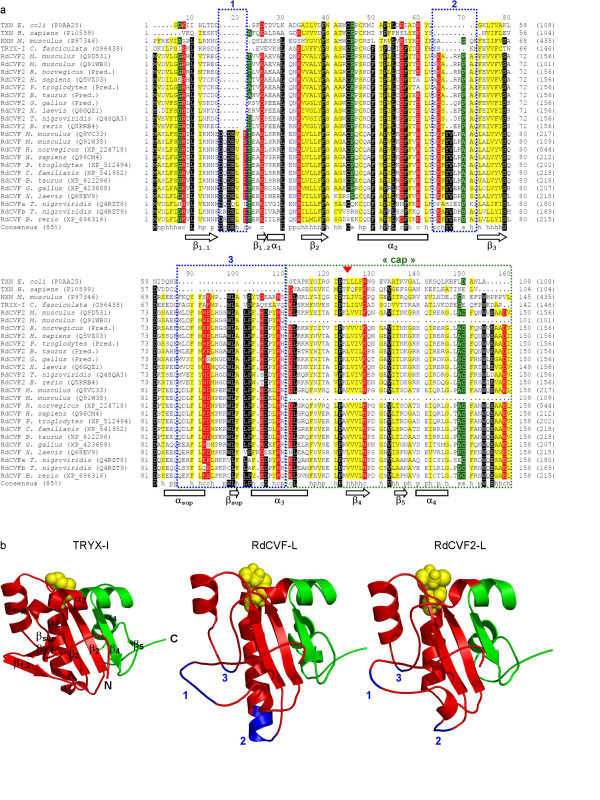
**Sequence and structure similarities of mouse RdCVF and RdCVF2 proteins with thioredoxin superfamily members**. Panel a shows the multiple sequence alignment of the thioredoxin (TXN) tryparedoxin (TRYX-I) nucleoredoxin (NXN) and the existing and predicted RdCVF and RdCVF2 proteins. The name, organism and accession number (in brackets) of each protein sequence are given (left). Identical (white text on black) small (A, D, G, P, S, T ; white text on green) hydrophobic (A, C, F, G, I, L, M, S, T, V, W, Y ; black text on yellow) polar (D, E, H, K, N, Q, R, S ; blue text) and charged (D, E, K, R ; white text on red) conserved residues are shown according to a conservation threshold of 85%. A consensus sequence is given below the multiple alignment in which s, h, p and c correspond to small, hydrophobic, polar and charged residues respectively. The secondary structures (β sheet and α helix) of the Crithidia fasciculata tryparedoxin I structure (1EWX) are given below the consensus sequence. The blue dashed rectangles indicate the three RdCVF(2) specific insertions. The green dashed rectangle shows the "cap" region absent in RdCVF(2)-S. The position of the human thioredoxin cleavage product (TRX80) is indicated (red triangle). Panel b displays the structure of the *Crithidia fasciculata *TRYX-I (1EWX) (left) mouse RdCVF-L (center) and mouse RdCVF2-L (right) models. Regions of TRYX-I backbone conserved in RdCVF(2)-L are colored in red. The "cap" region and the three specific insertions are depicted in green and blue respectively. The putative catalytic site (C_44_XXC_47_) is shown in yellow with a space-filling representation.

Proteins orthologous to RdCVF(-L/2-L) referring to the long isoforms of both RdCVF genes, were identified or predicted in vertebrates (*Rattus norvegicus*, *Homo sapiens*, *Pan troglodytes*, *Bos taurus*, *Canis familiaris*, *Gallus gallus*, *Xenopus laevis*, *Tetraodon nigroviridis*, *Brachydanio rerio*) according to protein or genome database searches. We aligned protein sequences of RdCVF, RdCVF2, tryparedoxin (TRYX), nucleoredoxin (NXN) and thioredoxin (TXN) (Figure 2, panel a). As exemplified by a phylogenetic analysis [see Additional file 2] among the TXN superfamily, RdCVF and RdCVF2 proteins are closely related to the TRYX and NXN members [20-25]. Even distant homologs such as *Crithidia fasciculata *tryparedoxin I (O96438, TRYX-I) [22] exhibit 42.5% and 45.4% sequence similarity to mouse RdCVF(-L/2-L) proteins. Three insertions in the multiple alignment (called 1,2 and 3) allow one to distinguish these phylogenetic protein families (Figure 2, panel a). Insertion 3 (residues 87–110) contains the conserved motif WLALP [W_108_(L, V)(A, F)(L, V, I)P_112_] and clearly discriminates the TRYX family [TRYX, NXN, RdCVF and RdCVF2] from TXN superfamily. Insertion 2 (63–72) and two additional residues (96–97) of insertion 3 allow one to differentiate the RdCVF and RdCVF2 proteins from the rest of the TRYX family. Finally, insertion 1 (16–21) unambiguously separates RdCVF from all the other TXN superfamily members including RdCVF2. Note that the thioredoxin active site C_44_XXC_47 _is only conserved in 44.4% (4/9) and 72.7% (8/11) of the RdCVF and RdCVF2 vertebrate proteins respectively.

### Structural modeling of RdCVF and RdCVF2

The high sequence similarity of RdCVFs with TRYX proteins prompted us to build the RdCVF(-L/2-L/-S/2-S) structural models with *Crithidia fasciculata *TRYX-I crystal structure (PDB accession number: 1EWX, 1.7 Å resolution structure) [22] as a template. By analogy with human TXN and TRX80 models [13] the RdCVF(-S/2-S) structure models were assumed to maintain the same overall folding. The TRYX-I (1EWX) and RdCVF(-L/2-L) structures are shown in Figure 2 panel b.

**Figure 3:**
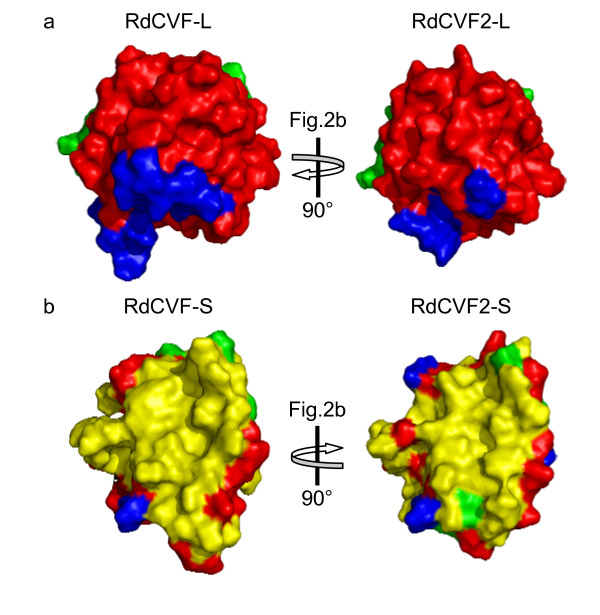
**Spatial proximity of the three specific insertions and specifically-accessible hydrophobic surface in the RdCVF-S and RdCVF2-S proteins**. Panel a displays the space-filling representation of RdCVF-L (left) and RdCVF2-L (right) using the same color code as Figure 2 panel b. Importantly, the RdCVF(2)-L models were rotated as indicated to focus on the three specific insertions. Note that catalytic site and "cap" region are on opposite ends of the protein. Panel b shows the RdCVF-S and RdCVF2-S models. Hydrophobic, small, polar and charged residues are colored in yellow, green, blue and red respectively. The models were rotated to focus on the large hydrophobic surface specifically-accessible in the short isoforms.

Figure 2 displays the 1EWX secondary structures (β-sheet and α-helix) below the multiple alignment (panel a) and in the TRYX-I 3D-structure (panel b). The insertions 1, 2 and 3 correspond respectively to: an increase in size of the β_1.1_-β_1.2 _sheets, a one turn extension in the α_2 _helix, and a larger structural region containing the TRYX-specific α_sup_-β_sup _and α_3 _extension. The two residues (96–97) belonging to insertion 3 in the RdCVF proteins correspond to a larger constrained loop before strand β_sup _and allow one to discriminate these proteins from TRYX members. It is worth noting that the location on the folded protein where the three insertions co-localize are on the opposite side from the putative catalytic site (C_44_XXC_47_) in RdCVFs (Figure 2, panel b). Finally, the C-terminal region absent in RdCVF(-S/2-S) proteins (hereafter called "cap" and depicted in green in Figure 2, panel b) is positionally fixed relative to the catalytic site. The "cap" region in TXN proteins interacts with the recycling enzyme thioredoxin reductase [7,13] and its absence might impair the thioredoxin activity in TRX80 and RdCVF(-S/2-S) [4,13]. A striking feature of these structural models is the clear spatial proximity of residues from the three insertions (Figure 3 panel a). This coincidence points to a possibly novel interaction site in RdCVF(-L/2-L). As expected, the backbone conformation of the refined model of RdCVF(-S/2-S) (shown in Figure 3, panel b) is the same as its counterpart in the long forms, with minor modifications observed in the side-chains at the interface between the non-"cap" and "cap" regions. It should be emphasized that the absence of the "cap" yields to the emergence of a major hydrophobic patch at the RdCVF(-S/2-S) surface (Figure 3, panel b). As a consequence the hydrophobic part of the accessible surface area of RdCVF proteins increases from 2394 Å ^2 ^in the long form to 3157 Å ^2 ^in the short form.

### RdCVF-S and RdCVF2-S are expressed in the retina in a rod-dependent manner

**Figure 4:**
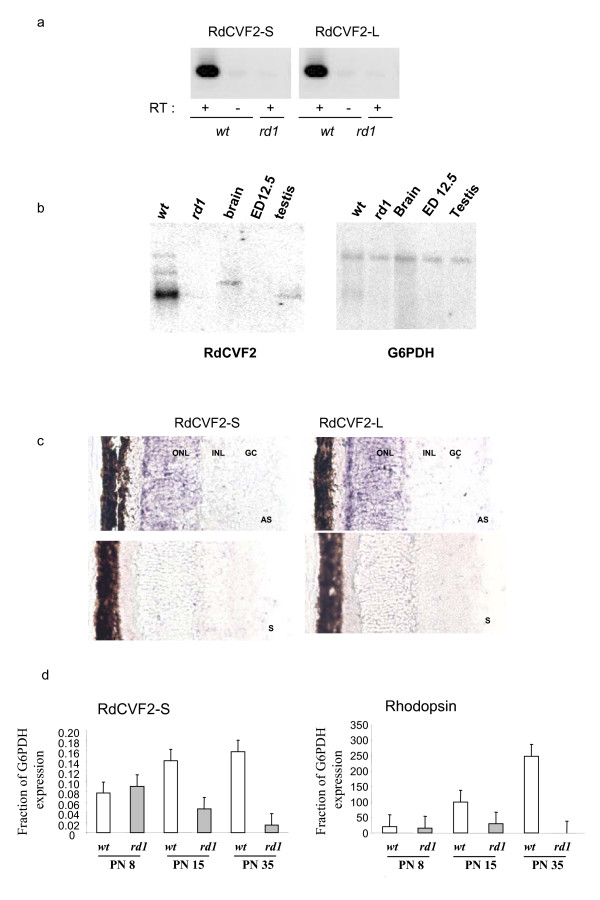
**Validation of the RdCVF2 expression in retina**. Panel a shows the expression of the short and long forms of RdCVF2 mRNA in the wild-type and *rd1 *retina at post-natal day 35. The RT-PCR product lengths are (RdCVF2-S: 176 bp, RdCVF2-L: 170 bp. Panel b: northern blotting analysis of RdCVF2 'exon 1) and G6PDH control. Panel c: *In situ *hybridization on sections of wild-type and *rd1 *mice retina with digoxigenin-labeled RdCVF2-S and L riboprobes (AS : antisens, S : sens). Panel d shows expression time-courses for both isoforms of RdCVF2 and rhodopsin in wild-type and *rd1 *mice during the first postnatal month. GC, Ganglion cells; INL, inner nuclear layer; ONL, outer nuclear layer. Original magnification : 40 ×.

We have measured the expression of RdCVF2(-S/-L) by RT-PCR. With primers amplifying the short (176 bp) and long (170 bp) isoforms, we found that RdCVF2-S and -L are expressed in the wild-type mouse retina (Figure 4, panel a). Interestingly, RdCVF2-S and -L expression was absent in the retina of the *rd1 *mouse after rod-photoreceptor degeneration. We have also studied the expression of RdCVF2 using northern blotting (Figure 4, panel b). In addition to the expression in the retina, most likely by rod photoreceptors since its expression is absent in the degenerated retina (*rd1*), we found weaker expression of RdCVF2 in the brain and testis. No expression was detected in the whole mouse embryo at embryonic day 12.5. To determine the expression of genes encoding RdCVF2-S and L across the retina we used *in situ *hybridization. Transcripts for RdCVF2-S and L were detected in the photoreceptor layer. No staining was observed with the sense control probes, supporting the specificity of the RdCVF2-S and L probes (Figure 4, panel c). No expression was detected in the *rd1 *retina after rod degeneration (result not shown).

We next analyzed the expression of RdCVF2-S during the process of rod degeneration (Figure 4, panel d). At post-natal day 8 (PN8) before the onset of rod loss, RdCVF2-S is expressed at similar level in the wild-type and in the *rd1 *retina similarly to the rod photopigment gene rhodopsin. From PN15 to PN35 the degeneration of rods (measured by the decrease in rhodopsin expression) is correlated with a decrease in RdCVF2-S expression. These results indicate that RdCVF2-S is expressed in a rod-dependent manner [see Additional file 3].

### RdCVF2 mRNA is not only expressed in the retina but also in other tissues

We searched in the EMBL public database for mouse EST and mRNA sequences corresponding to the RdCVF(-L/-S/2-L/2-S) mRNAs to estimate the tissue distribution of each isoform [see Additional file 4]. As reported before [4] RdCVF-L and RdCVF-S mRNAs are specifically expressed in eye and retina as 20/23 and 4/4 sequences were found in these tissues respectively. The mouse RdCVF2-L mRNA is preferentially expressed in retina (10/24) but is also present in other tissue types such as tumor (2) testis (2) stem cells (2) amnion (1) placenta (1) oviduct (1) fetus (1) thymus (1) and mammary gland (1). Finally, EST and mRNA sequences corresponding to RdCVF2-S are exclusively expressed in retina (3/4). We were able to detect the expression of RdCVF2 in the testis and brain (Figure 4, panel b).

### RdCVF2-S cone viability effects

**Figure 5:**
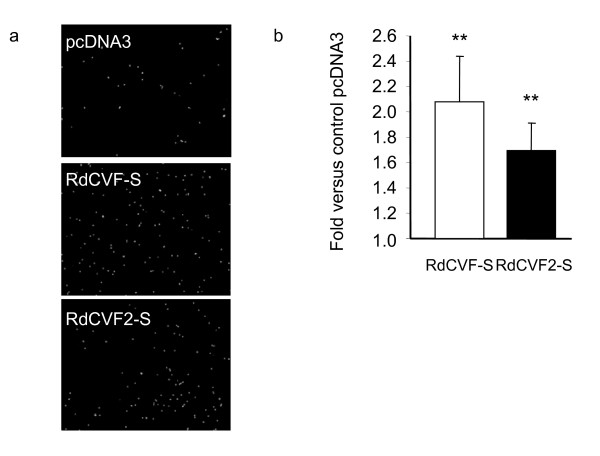
**Cone viability assay of RdCVF-S and RdCVF2-S**. Panel a displays cells from cone-enriched cultures labeled with the viability dye calcein-AM and incubated with conditioned media from COS-1 cells transfected with: empty vector pcDNA3, pcDNA-RdCVF-S or pcDNA-RdCVF2-S. Panel b shows the rescue activity of RdCVF-S and RdCVF2-S when compared to that of empty vector. Statistical analysis (Tuckey test) shows that the results are statistically significant (p < 0.001).

The strong similarities between RdCVF and RdCVF2 loci in terms of gene organization, conservation of sequence and rod-dependent expression led us to hypothesize that RdCVF2-S protein might also be able to promote cone viability as previously reported for RdCVF-S [4]. Indeed, the figure 5 panel a and b shows that the number of live cells in the presence of RdCVF-S is twice as the control (pcDNA3). A less pronounced, but statistically significant, increase in cone viability (1.6 fold) is observed for RdCVF2-S. These findings confirm that RdCVF2-S is also a cone viability factor similar to RdCVF-S [4]. Importantly, no synergistic trophic effect on cones is observed when both RdCVF-S and RdCVF2-S are co-tranfected in COS-1 cells pointing to use of the same pathway by both factors (data not shown).

## Discussion

### Identification of RdCVF2

In this paper, we report the identification of a gene paralogous to RdCVF called RdCVF2. The analysis of gene structure conservation and the EST database searches with experimental validation by RT-PCR and *in situ *hybridization indicate two alternative transcripts from both genes, expressed in a rod-dependent manner and translated into the RdCVF(-S/2-S) and RdCVF(-L/2-L) short and long protein isoforms. A phylogenetic analysis suggests that both genes are strongly conserved in most vertebrates. Experimental validations demonstrate a cone viability activity of RdCVF2-S that is similar to the one of RdCVF. Taken together, these genes define a novel bifunctional gene family expressed in vertebrate retinas with trophic activity and significant potential as therapeutic targets in human retinal diseases.

### Bifunctional activity of the RdCVF family members

Like thioredoxin, the RdCVF family is bifunctional and pleiotropic in vertebrates. The short isoform exerts a trophic activity while the long isoform's function is unknown, but presumably involves a redox activity. RdCVF and RdCVF2 proteins share high sequence and structure homologies with tryparedoxin, a member of the thioredoxin family that might be of functional significance. The non-conservation of the C_44_XXC_47 _catalytic site implies that the thiol-oxydoreductase activity of the long isoforms in RdCVFs may have become dispensable. Nevertheless, it is noteworthy that the cysteins of the catalytic domain are never lost in both RdCVF-L and RdCVF2-L proteins in the same organism.

As it has been previously published [4], our current results contribute to explain the lack of thiol-oxydoreductase activity in the short isoforms of the RdCVFs. Since the "cap" region directly interacts with the thioredoxin reductase which recycles the enzyme activity [7,13], its absence in RdCVF-S and RdCVF2-S proteins prevent them from such a redox function.

### RdCVF-S and RdCVF2-S cone viability and signaling mechanisms

Analysis of the RdCVF-S and RdCVF2-S structural models provided evidence for two features. First, since the short isoforms of the RdCVFs lack the C-terminal "cap" region of the long isoforms, and have no thiol-oxydoreductase activity, they expose a large accessible hydrophobic patch (Figure 3, panel b). This patch may be where these proteins interact with other proteins or cell membrane structures.

Second, the three RdCVF-specific insertions (Figure 2, panel b) all co-localize at the opposite pole from the catalytic site. This novel surface feature may also constitute an interaction site with a cell-surface receptor expressed by cone photoreceptors. Mutation analyses will prove useful in exploring the roles of these two features. Since thioredoxin are secreted by a pathway that does not require leader sequence [26], it is also theoretically possible that a putative receptor is present within the cytoplasm of cones and that RdCVF-S and RdCVF2-S are diffused through the interphotoreceptor matrix penetrating photoreceptor cells.

RdCVF-S was demonstrated to be involved with cone viability by a 60% reduction in the rescue activity of conditioned media upon rod-enriched retinal explants after immunodepletion with anti-RdCVF-S antibodies [4]. The similarity between the two factors suggests that they belong to similar signaling pathways. Therefore, RdCVF2-S might be responsible for the remaining 40% cone viability activity. Similar experiments using antibodies for both genes would help to determine whether the trophic activity can be full accounted for by these two proteins. Co-immunoprecipitation would also be interesting since the short isoforms bear potential interaction domains which imply binding partners. Indeed, by analogy with TRX80 that dimerizes in solution [13] the very large hydrophobic surface created by the "cap" removal of RdCVF-L and RdCVF2-L (Figure 3, panel b) may promote homodimerization or heterodimerization among RdCVF-S and RdCVF2-S.

Photoreceptors constitute the cells with the highest rate of oxidative metabolism in the body. As the outer retina (the photoreceptor layer) is avascular, the oxygen is provided by the high blood flow from the underlying choroid. Since this blood flow is not regulated by oxygen consumption, primary rod (97% of all photoreceptors in the mouse retina) degeneration leads to a huge increase in oxygen [27,28]. As thioredoxin enzymes participate in redox homeostasis [29], the RdCVF gene may have originally served an extra thiol-oxydoreductase activity to prevent damage linked to hyperoxia of photoreceptors resulting from light. One could suppose that the RdCVF family bifunctionality might form a regulatory loop in which the long form senses oxygen levels and transfer this signal to the short form that would exert a trophic effect on neighboring cells and would maintain a correct cell-oxygen ratio.

## Conclusion

By data mining using RdCVF sequence, we have identified a novel trophic factor for cone survival. This second factor defines a novel family of bifunctional proteins with potential involvement in neuroprotection and response to oxidative stress. The homology of both factors with the thioredoxin family suggests that the RdCVF family derives from an ancestor thioredoxin gene that has been recruited during evolution to serve the protection of cone photoreceptors.

## Methods

### Database searches

The UCSC genome browser BLAT [19,30] server was used to map the mouse RdCVF and RdCVF2 genes to all the available vertebrate genomes and to extract the corresponding genomic sequences. In order to identify candidate RdCVF and RdCVF2 orthologous proteins, homology searches in the UniProt [6] and EMBL [31] public sequence databases were performed using the BLAST programs [32,33]. Mouse mRNA and EST sequences associated with both RdCVF and RdCVF2 isoforms (L and S) were used to estimate the tissue specificity of each messenger.

### Multiple alignments of DNA and protein sequences

TBA [34] and PipeAlign [35] programs were used with default parameters to generate the multiple alignments of genomic and protein sequences respectively. Protein alignment occasionally included manual adjustments in keeping with the protein secondary structure conservation.

### Phylogenetic tree of the RdCVF family

The PhyloWin program [36] was used to generate the phylogenetic tree based on the multiple alignment of protein sequences using the neighbour joining reconstruction algorithm with pairwise gap removal and 500 bootstrap replicates. Only the first 155 and 147 residues of RdCVF-L and RdCVF2-L proteins respectively were used.

### Structural modeling of the short and long RdCVF and RdCVF2 variants

Structural models for mouse RdCVF and RdCVF2 (both S and L forms) using the 155 and 147 first residues respectively were constructed using the Builder homology modeling package [37-39]. The final models were further refined by energy minimization, using ENCAD [40]. On each model 1000 steps of conjugate gradient minimization was applied. The E_146_(1EWX) → P_146_(RdCVF-L) mutation obliges the local backbone conformation in the template structure to be adapted to fit the proline (Figure 2, panel a and b). Builder samples simultaneously the conformation of the loops in the five insertions/deletions and in the E → P mutation region, and the conformation of the side-chains, using a self consistent mean field approach. PyMOL [41] was used to render the final structures.

### Real-time RT-PCR and Northern blotting

Total RNA from neural retina of 8, 15 and 35-day-old wild type (C57BL/6@N), rd1 mutant (C3H/He@N) mice was purified by cesium gradient [42]. Double-stranded cDNA was synthesized from 5 μg total RNA using Superscript Choice System (Invitrogen, Carlsbad, CA). cDNAs were produced by random priming and normalized according to glucose-6-phosphate dehydrogenase mRNA. First strand cDNA (0.2 μl) was amplified in triplicate using 2 μM of the specific primers. Primers 5'- CATCACCAACAAAGGGCGGAAG -3' and 5'- CATTCCTCAGCAGAGAAGGGAAC -3' were used for RdCVF2-S; primers 5'- CCGTGCTATTGTTTCAGAGCCCTTAACTTTCTATC -3' and 5'- CTGACACTCCAATCGTAAAAGGCAGAAAACGC -3' were used for RdCVF2-L. Primers 5'-AAGCCGATGAGCAACTTCC-3'; 5'-TCATCTCCCAGTGGATTCTT-3' were used for rhodopsin ; 5'-GCAGTCACCAAGAACATTCAAG -3' 5'-CCCAAATTCATCAAAATAGCCC-3' were used for G6PDH on a lightcycler (Roche, Basel, Switzerland).

The absence of DNA contamination was checked by omitting the reverse transcriptase. Results are displayed as fold difference compared to the lowest expression.

For northern blotting analysis, 2 μg of poly-A mRNA was used and the membrane was hybridized to a probe corresponding to exon 1 of the RdCVF2 gene using standard method.

### In situ hybridization

The expression of RdCVF2-S and -L mRNA in the retina was analyzed by in situ hybridization with a digoxigenin-labeled murine antisense riboprobe.

After defrosting and drying at room temperature, sections were post-fixed on ice for 10 min in 4% paraformaldehyde washed in PBS at room temperature for 10 min.

Mouse RdCVF2-S and RdCVF2-L was amplified by PCR using the following primers: primers 5'-GTAGCTTTGTACTTTGCGGCG-3' and 5'-GTCATCAGAAAATGTATCACCTCCATAGG-3' for RdCVF2-S; primers 5'-GCCATCTCTGCGACTTATTTTTACC-3' and 5'-AATTAGTGCCACCAGCACCATC-3' for RdCVF2-L.

The PCR product was cloned into PGEM easy vector (Promega, France).

Sections were hybridized with sense and antisense RdCVF2 mRNA probes generated from SP6 or T7 promoters and labeled with digoxigenin-UTP (Boehringer, Mannheim, Germany). *In situ *hybridization and digoxigenin-labeled probe detection were performed as described previously [43]. The specificity of the staining was demonstrated by the lack of hybridization signal with the sense probe.

### Cone viability assay

RdCVF(-S/2-S) isoforms were cloned into the expression plasmid pcDNA3 and transfected into COS-1 cells. 48 hours after transfection, the conditioned media from the COS-transfected cells was harvested and incubated with a cone-enriched primary cell culture system from chicken embryo (60–80% of cones) [44]. After seven days of incubation, a period over which these post-mitotic cells degenerate, the viability of the cells in the culture was scored using the Live/Dead assay (Molecular probes, Eugene, OR) and a cell counting platform as previously described [4]. The viability corresponding to three independent assays is represented as fold over pcDNA3 used as negative control.

## Authors' contributions

FC participated in the design and the coordination of the study, participated in the bioinformatics analysis (protein and genome sequence alignments, structural modelling) and drafted the manuscript. TL conceived of the study, and participated in its design and coordination and drafted the manuscript. CJ carried out the in situ hybridization and helped to draft the manuscript. AL participated in the protein and genome sequence alignments, helped to draft the manuscript. NB participated in the protein and genome sequence alignments. PK participated in the structural modelling. GL and ELB carried out the RT-PCR. AH revised the manuscript for important intellectual content. JAS participated in the design of the study and helped to draft the manuscript. OP coordinated the design of the study, participated in the protein sequence alignment and helped to draft the manuscript.

## Supplementary Material

Additional file 1 **Gene structure and exon coordinates of RdCVF and RdCVF2 genes**. The table reports the exon positions of the RdCVF(2) isoforms on 13 vertebrate genomes. The genome versions are indicated at the right. Click here for file

Additional file 2 **Phylogenetic tree of the RdCVF and RdCVF2 proteins**. The phylogenetic tree based on the multiple alignment displayed in Figure 2 panel a was done using PhyloWin. The name, organism and accession number are given for each protein. Bootstrap values are indicated at nodes. Click here for file

Additional file 3 **Time course expression of RdCVF2 and Rhodopsin**. Both graphs correspond respectively to RdCVF2 and Rhodopsin prosets. The wt and rd1 are on the same genetic background (C3H). Click here for file

Additional file 4 **Tissue distribution of RdCVF and RdCVF2 mRNAs**. The table describes tissue expression of the EST and mRNA sequences available in the EMBL databases corresponding to RdCVF(-L/-S/2-L/2-S) transcripts. Click here for file

## References

[B1] Carter-Dawson LD, LaVail MM, Sidman RL (1978). Differential effect of the rd mutation on rods and cones in the mouse retina. Invest Ophthalmol Vis Sci.

[B2] Portera-Cailliau C, Sung CH, Nathans J, Adler R (1994). Apoptotic photoreceptor cell death in mouse models of retinitis pigmentosa. Proc Natl Acad Sci U S A.

[B3] Mohand-Said S, Deudon-Combe A, Hicks D, Simonutti M, Forster V, Fintz AC, Leveillard T, Dreyfus H, Sahel JA (1998). Normal retina releases a diffusible factor stimulating cone survival in the retinal degeneration mouse. Proc Natl Acad Sci U S A.

[B4] Leveillard T, Mohand-Said S, Lorentz O, Hicks D, Fintz AC, Clerin E, Simonutti M, Forster V, Cavusoglu N, Chalmel F, Dolle P, Poch O, Lambrou G, Sahel JA (2004). Identification and characterization of rod-derived cone viability factor. Nat Genet.

[B5] Sahel JA (2005). Saving cone cells in hereditary rod diseases: a possible role for rod-derived cone viability factor (RdCVF) therapy. Retina.

[B6] Wu CH, Apweiler R, Bairoch A, Natale DA, Barker WC, Boeckmann B, Ferro S, Gasteiger E, Huang H, Lopez R, Magrane M, Martin MJ, Mazumder R, O'Donovan C, Redaschi N, Suzek B (2006). The Universal Protein Resource (UniProt): an expanding universe of protein information. Nucleic Acids Res.

[B7] Holmgren A (1985). Thioredoxin. Annu Rev Biochem.

[B8] Holmgren A (1989). Thioredoxin and glutaredoxin systems. J Biol Chem.

[B9] Arner ES, Holmgren A (2000). Physiological functions of thioredoxin and thioredoxin reductase. Eur J Biochem.

[B10] Powis G, Montfort WR (2001). Properties and biological activities of thioredoxins. Annu Rev Pharmacol Toxicol.

[B11] Holmgren A (1979). Reduction of disulfides by thioredoxin. Exceptional reactivity of insulin and suggested functions of thioredoxin in mechanism of hormone action. J Biol Chem.

[B12] Holmgren A (1979). Thioredoxin catalyzes the reduction of insulin disulfides by dithiothreitol and dihydrolipoamide. J Biol Chem.

[B13] Pekkari K, Gurunath R, Arner ES, Holmgren A (2000). Truncated thioredoxin is a mitogenic cytokine for resting human peripheral blood mononuclear cells and is present in human plasma. J Biol Chem.

[B14] Pekkari K, Goodarzi MT, Scheynius A, Holmgren A, Avila-Carino J (2005). Truncated thioredoxin (Trx80) induces differentiation of human CD14+ monocytes into a novel cell type (TAMs) via activation of the MAP kinases p38, ERK, and JNK. Blood.

[B15] Liu A, Arbiser JL, Holmgren A, Klein G, Klein E (2005). PSK and Trx80 inhibit B-cell growth in EBV-infected cord blood mononuclear cells through T cells activated by the monocyte products IL-15 and IL-12. Blood.

[B16] Pekkari K, Avila-Carino J, Gurunath R, Bengtsson A, Scheynius A, Holmgren A (2003). Truncated thioredoxin (Trx80) exerts unique mitogenic cytokine effects via a mechanism independent of thiol oxido-reductase activity. FEBS Lett.

[B17] Jeffery CJ (1999). Moonlighting proteins. Trends Biochem Sci.

[B18] Jeffery CJ (2003). Moonlighting proteins: old proteins learning new tricks. Trends Genet.

[B19] Hinrichs AS, Karolchik D, Baertsch R, Barber GP, Bejerano G, Clawson H, Diekhans M, Furey TS, Harte RA, Hsu F, Hillman-Jackson J, Kuhn RM, Pedersen JS, Pohl A, Raney BJ, Rosenbloom KR, Siepel A, Smith KE, Sugnet CW, Sultan-Qurraie A, Thomas DJ, Trumbower H, Weber RJ, Weirauch M, Zweig AS, Haussler D, Kent WJ (2006). The UCSC Genome Browser Database: update 2006. Nucleic Acids Res.

[B20] Micossi E, Hunter WN, Leonard GA (2002). De novo phasing of two crystal forms of tryparedoxin II using the anomalous scattering from S atoms: a combination of small signal and medium resolution reveals this to be a general tool for solving protein crystal structures. Acta Crystallogr D Biol Crystallogr.

[B21] Krumme D, Budde H, Hecht HJ, Menge U, Ohlenschlager O, Ross A, Wissing J, Wray V, Flohe L (2003). NMR studies of the interaction of tryparedoxin with redox-inactive substrate homologues. Biochemistry.

[B22] Alphey MS, Leonard GA, Gourley DG, Tetaud E, Fairlamb AH, Hunter WN (1999). The high resolution crystal structure of recombinant Crithidia fasciculata tryparedoxin-I. J Biol Chem.

[B23] Eklund H, Gleason FK, Holmgren A (1991). Structural and functional relations among thioredoxins of different species. Proteins.

[B24] Kurooka H, Kato K, Minoguchi S, Takahashi Y, Ikeda J, Habu S, Osawa N, Buchberg AM, Moriwaki K, Shisa H, Honjo T (1997). Cloning and characterization of the nucleoredoxin gene that encodes a novel nuclear protein related to thioredoxin. Genomics.

[B25] Laughner BJ, Sehnke PC, Ferl RJ (1998). A novel nuclear member of the thioredoxin superfamily. Plant Physiol.

[B26] Nickel W (2003). The mystery of nonclassical protein secretion. A current view on cargo proteins and potential export routes. Eur J Biochem.

[B27] Travis GH, Groshan KR, Lloyd M, Bok D (1992). Complete rescue of photoreceptor dysplasia and degeneration in transgenic retinal degeneration slow (rds) mice. Neuron.

[B28] Stone J, Maslim J, Valter-Kocsi K, Mervin K, Bowers F, Chu Y, Barnett N, Provis J, Lewis G, Fisher SK, Bisti S, Gargini C, Cervetto L, Merin S, Peer J (1999). Mechanisms of photoreceptor death and survival in mammalian retina. Prog Retin Eye Res.

[B29] Nakamura H (2004). Thioredoxin as a key molecule in redox signaling. Antioxid Redox Signal.

[B30] Kent WJ (2002). BLAT--the BLAST-like alignment tool. Genome Res.

[B31] Cochrane G, Aldebert P, Althorpe N, Andersson M, Baker W, Baldwin A, Bates K, Bhattacharyya S, Browne P, van den Broek A, Castro M, Duggan K, Eberhardt R, Faruque N, Gamble J, Kanz C, Kulikova T, Lee C, Leinonen R, Lin Q, Lombard V, Lopez R, McHale M, McWilliam H, Mukherjee G, Nardone F, Pastor MP, Sobhany S, Stoehr P, Tzouvara K, Vaughan R, Wu D, Zhu W, Apweiler R (2006). EMBL Nucleotide Sequence Database: developments in 2005. Nucleic Acids Res.

[B32] Altschul SF, Gish W, Miller W, Myers EW, Lipman DJ (1990). Basic local alignment search tool. J Mol Biol.

[B33] Altschul SF, Madden TL, Schaffer AA, Zhang J, Zhang Z, Miller W, Lipman DJ (1997). Gapped BLAST and PSI-BLAST: a new generation of protein database search programs. Nucleic Acids Res.

[B34] Blanchette M, Kent WJ, Riemer C, Elnitski L, Smit AF, Roskin KM, Baertsch R, Rosenbloom K, Clawson H, Green ED, Haussler D, Miller W (2004). Aligning multiple genomic sequences with the threaded blockset aligner. Genome Res.

[B35] Plewniak F, Bianchetti L, Brelivet Y, Carles A, Chalmel F, Lecompte O, Mochel T, Moulinier L, Muller A, Muller J, Prigent V, Ripp R, Thierry JC, Thompson JD, Wicker N, Poch O (2003). PipeAlign: A new toolkit for protein family analysis. Nucleic Acids Res.

[B36] Galtier N, Gouy M, Gautier C (1996). SEAVIEW and PHYLO_WIN: two graphic tools for sequence alignment and molecular phylogeny. Comput Appl Biosci.

[B37] Koehl P, Delarue M (1994). Application of a self-consistent mean field theory to predict protein side-chains conformation and estimate their conformational entropy. J Mol Biol.

[B38] Koehl P, Delarue M (1995). A self consistent mean field approach to simultaneous gap closure and side-chain positioning in homology modelling. Nat Struct Biol.

[B39] Koehl P, Delarue M (1996). Mean-field minimization methods for biological macromolecules. Curr Opin Struct Biol.

[B40] Levitt M, Hirshberg M, Sharon R, Daggett V (1995). Potential Energy Function and Parameters for Simulations of the Molecular Dynamics of Proteins and Nucleic Acids in Solution. Computer Physics Comm.

[B41] PyMOL. http://www.pymol.org.

[B42] Chirgwin JM, Przybyla AE, MacDonald RJ, Rutter WJ (1979). Isolation of biologically active ribonucleic acid from sources enriched in ribonuclease. Biochemistry.

[B43] Roger J, Brajeul V, Thomasseau S, Hienola A, Sahel JA, Guillonneau X, Goureau O (2006). Involvement of Pleiotrophin in CNTF-mediated differentiation of the late retinal progenitor cells. Dev Biol.

[B44] Fintz AC, Audo I, Hicks D, Mohand-Said S, Leveillard T, Sahel J (2003). Partial characterization of retina-derived cone neuroprotection in two culture models of photoreceptor degeneration. Invest Ophthalmol Vis Sci.

