# Supplementary material for: Rod-derived Cone Viability Factor-2 is a novel bifunctional-thioredoxin-like protein with therapeutic potential
Source: BMC Mol Biol. 2007 Aug 31;8:74. doi: 10.1186/1471-2199-8-74 (PMC2064930; doi:10.1186/1471-2199-8-74)
Supplement: Additional file 1 — Gene structure and exon coordinates of RdCVF and RdCVF2 genes. The table reports the exon positions of the RdCVF(2) isoforms on 13 vertebrate genomes. The genome versions are indicated at the right. [file 1471-2199-8-74-S1.ppt]

## Slide 1
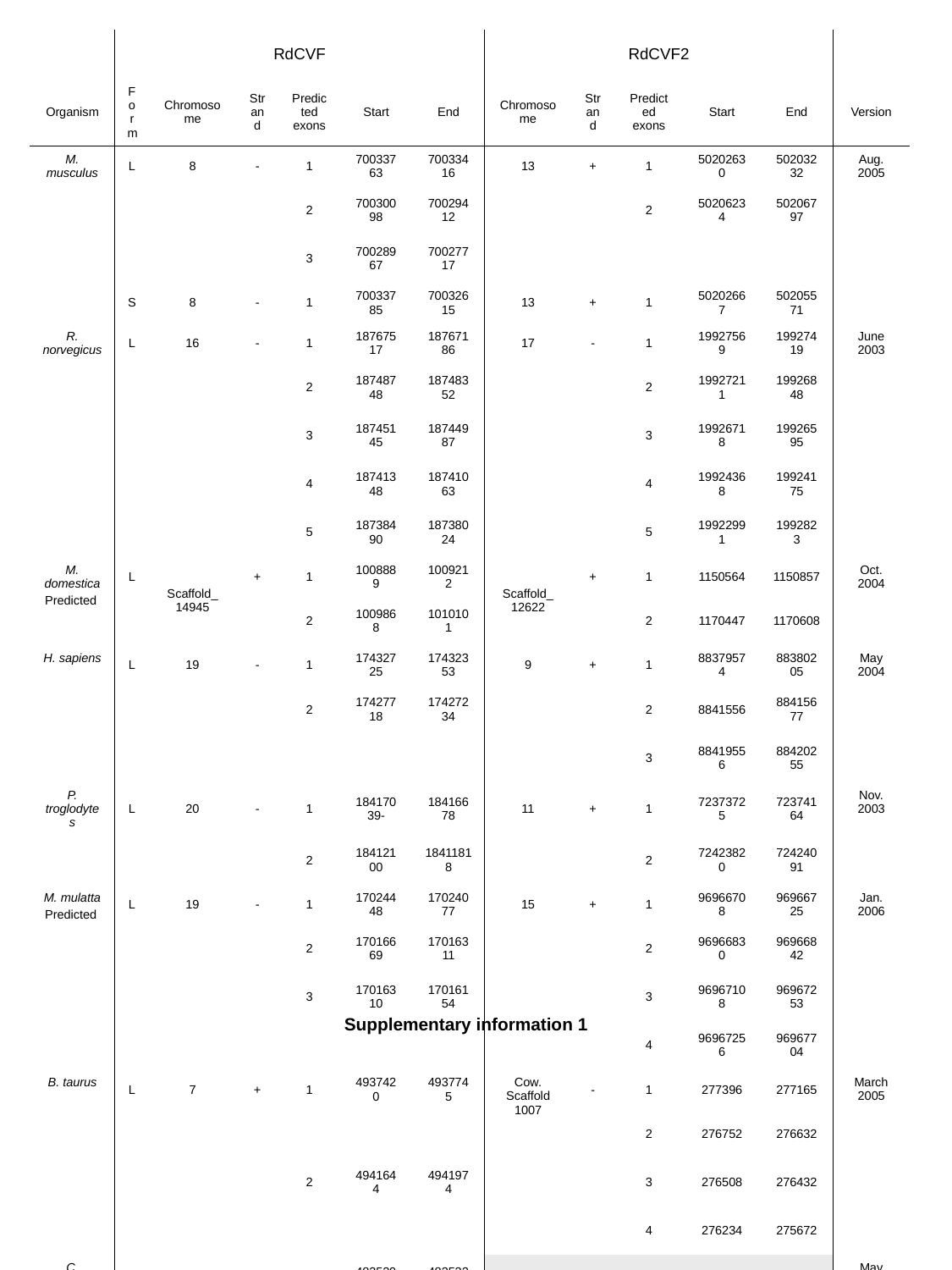

| | RdCVF | | | | | | RdCVF2 | | | | | |
| --- | --- | --- | --- | --- | --- | --- | --- | --- | --- | --- | --- | --- |
| Organism | Form | Chromosome | Strand | Predicted exons | Start | End | Chromosome | Strand | Predicted exons | Start | End | Version |
| M. musculus | L | 8 | - | 1 | 70033763 | 70033416 | 13 | + | 1 | 50202630 | 50203232 | Aug. 2005 |
| | | | | 2 | 70030098 | 70029412 | | | 2 | 50206234 | 50206797 | |
| | | | | 3 | 70028967 | 70027717 | | | | | | |
| | S | 8 | - | 1 | 70033785 | 70032615 | 13 | + | 1 | 50202667 | 50205571 | |
| R. norvegicus | L | 16 | - | 1 | 18767517 | 18767186 | 17 | - | 1 | 19927569 | 19927419 | June 2003 |
| | | | | 2 | 18748748 | 18748352 | | | 2 | 19927211 | 19926848 | |
| | | | | 3 | 18745145 | 18744987 | | | 3 | 19926718 | 19926595 | |
| | | | | 4 | 18741348 | 18741063 | | | 4 | 19924368 | 19924175 | |
| | | | | 5 | 18738490 | 18738024 | | | 5 | 19922991 | 1992823 | |
| M. domestica Predicted | L | Scaffold\_ 14945 | + | 1 | 1008889 | 1009212 | Scaffold\_ 12622 | + | 1 | 1150564 | 1150857 | Oct. 2004 |
| | | | | 2 | 1009868 | 1010101 | | | 2 | 1170447 | 1170608 | |
| H. sapiens | L | 19 | - | 1 | 17432725 | 17432353 | 9 | + | 1 | 88379574 | 88380205 | May 2004 |
| | | | | 2 | 17427718 | 17427234 | | | 2 | 8841556 | 88415677 | |
| | | | | | | | | | 3 | 88419556 | 88420255 | |
| P. troglodytes | L | 20 | - | 1 | 18417039- | 18416678 | 11 | + | 1 | 72373725 | 72374164 | Nov. 2003 |
| | | | | 2 | 18412100 | 18411818 | | | 2 | 72423820 | 72424091 | |
| M. mulatta Predicted | L | 19 | - | 1 | 17024448 | 17024077 | 15 | + | 1 | 96966708 | 96966725 | Jan. 2006 |
| | | | | 2 | 17016669 | 17016311 | | | 2 | 96966830 | 96966842 | |
| | | | | 3 | 17016310 | 17016154 | | | 3 | 96967108 | 96967253 | |
| | | | | | | | | | 4 | 96967256 | 96967704 | |
| B. taurus | L | 7 | + | 1 | 4937420 | 4937745 | Cow. Scaffold 1007 | - | 1 | 277396 | 277165 | March 2005 |
| | | | | | | | | | 2 | 276752 | 276632 | |
| | | | | 2 | 4941644 | 4941974 | | | 3 | 276508 | 276432 | |
| | | | | | | | | | 4 | 276234 | 275672 | |
| C. familiaris | L | 20 | + | 1 | 48352944 | 48353269 | | | | | | May 2005 |
| | | | | 2 | 48355327 | 48355657 | | | | | | |
| G. gallus | L | UN | + | 1 | 123841185 | 123841510 | UN | + | 1 | 133738855 | 133739307 | Feb 2004 |
| | | | | 2 | 123842780 | 123843177 | | | 2 | 133739794 | 133740148 | |
| X. tropicalis | L | tropicalis. scaffold\_15 | - | 1 | 80461 | 80087 | Scaffold\_53 Tropicalis | - | 1 | 2334789 | 2334755 | Aug. 2005 |
| | | | | 2 | 76212 | 75894 | | | 2 | 2334735 | 2334728 | |
| | | | | 3 | 75866 | 75716 | | | 3 | 2334724 | 2334417 | |
| | | | | | | | | | 4 | 2330510 | 2330508 | |
| | | | | | | | | | 5 | 2330289 | 2330107 | |
| | | | | | | | | | 6 | 2330074 | 2330055 | |
| | | | | | | | | | 7 | 2329662 | 2329637 | |
| B. rerio | L | 1 | + | 1 | 61179797 | 61180122 | NA | + | 1 | 215875045 | 215875501 | June 2004 |
| | | | | 2 | 61181181 | 61181502 | | | 2 | 215877224 | 215877552 | |
| | | | | | | | | | 3 | 215878206 | 215878267 | |
| | | | | | | | | | 4 | 215878315 | 215878416 | |
| T. rubripes Predicted | La | Un | - | 1 | 96315137 | 96314904 | UN | - | 1 | 83958023 | 83957724 | Aug. 2002 |
| | | | | 2 | 96314799 | 96314707 | | | 2 | 83956987 | 83956829 | |
| | | | | 3 | 96314508 | 96314311 | | | | | | |
| | Lb | Un | - | 1 | 96312395 | 96312165 | | | | | | |
| | | | | 2 | 96312074 | 96311982 | | | | | | |
| | | | | 3 | 96311525 | 96311376 | | | | | | |
| T. nigroviridis | La | 18 | + | 1 | 1589517 | 1589750 | 4 | + | 1 | 3744208 | 3744507 | Feb 2004 |
| | | | | 2 | 1589826 | 1589918 | | | 2 | 3744942 | 3745106 | |
| | | | | 3 | 1590127 | 1590339 | | | | | | |
| | Lb | 18 | + | 1 | 1591515 | 1591748 | | | | | | |
| | | | | 2 | 1591859 | 1591948 | | | | | | |
| | | | | 3 | 1592094 | 1592216 | | | | | | |
Supplementary information 1
