# Supplementary figures and images for: Rod-derived Cone Viability Factor-2 is a novel bifunctional-thioredoxin-like protein with therapeutic potential
Source: BMC Mol Biol. 2007 Aug 31;8:74. doi: 10.1186/1471-2199-8-74 (PMC2064930; doi:10.1186/1471-2199-8-74)

## Slide 1
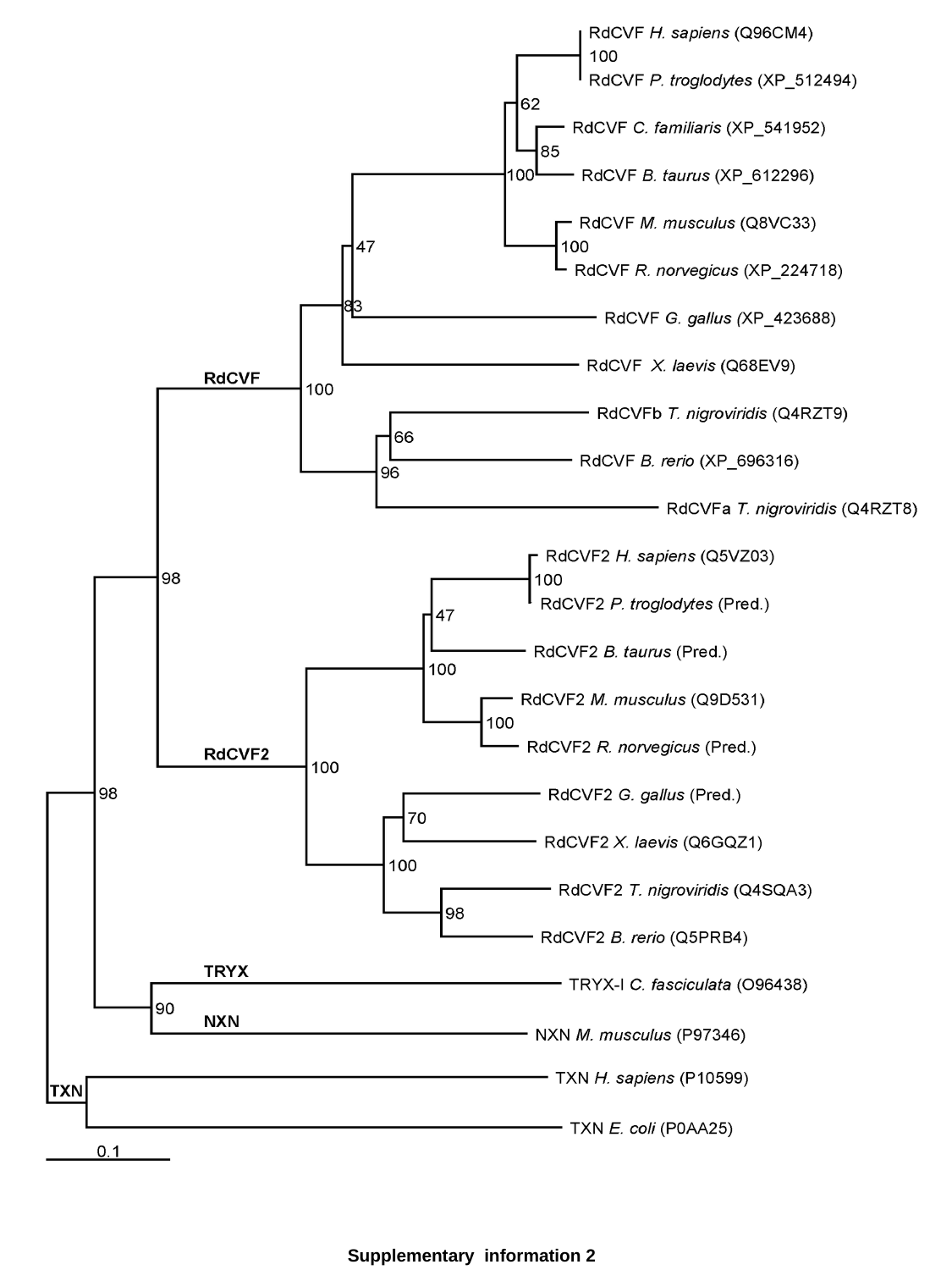

Supplementary information 2

Supplement: Additional file 2 — Phylogenetic tree of the RdCVF and RdCVF2 proteins. The phylogenetic tree based on the multiple alignment displayed in Figure 2 panel a was done using PhyloWin. The name, organism and accession number are given for each protein. Bootstrap values are indicated at nodes. [file 1471-2199-8-74-S2.ppt]
