# Supplementary material for: Rod-derived Cone Viability Factor-2 is a novel bifunctional-thioredoxin-like protein with therapeutic potential
Source: BMC Mol Biol. 2007 Aug 31;8:74. doi: 10.1186/1471-2199-8-74 (PMC2064930; doi:10.1186/1471-2199-8-74)
Supplement: Additional file 3 — Time course expression of RdCVF2 and Rhodopsin. Both graphs correspond respectively to RdCVF2 and Rhodopsin prosets. The wt and rd1 are on the same genetic background (C3H). [file 1471-2199-8-74-S3.pdf]

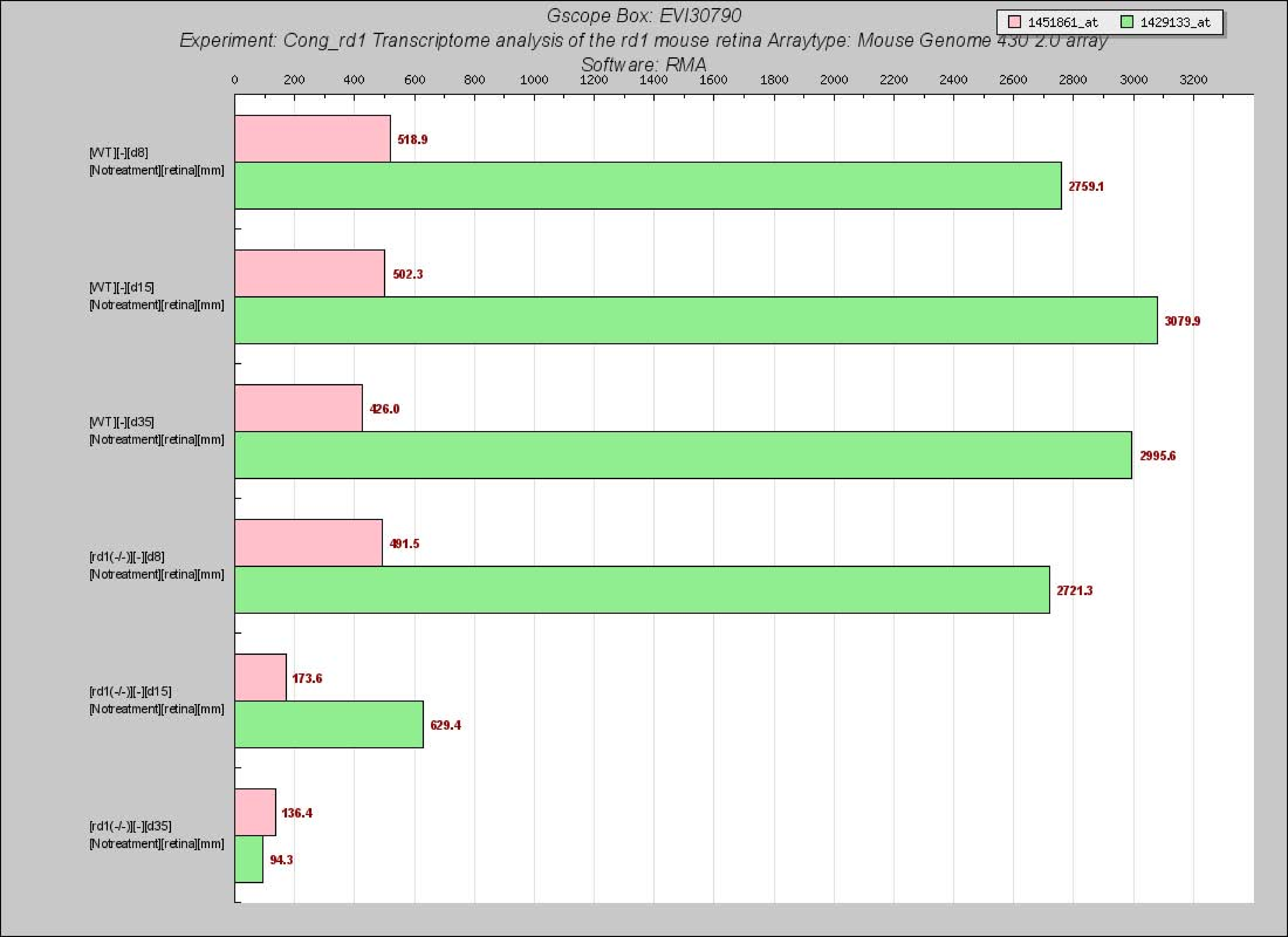

Gscope Box: EVI30790
Experiment: Cong_rd1 Transcriptome analysis of the rd1 mouse retina Arraytype: Mouse Genome 430 2.0 array
Software: RMA
1451861_at
1429133_at
0
200
400
600
800
1000
1200
1400
1600
1800
2000
2200
2400
2600
2800
3000
3200
[WT][-][d8] [Notreatment][retina][mm]
518.9
2759.1
[WT][-][d15] [Notreatment][retina][mm]
502.3
3079.9
[WT][-][d35] [Notreatment][retina][mm]
426.0
2995.6
[rd1(-/-)][-][d8] [Notreatment][retina][mm]
491.5
2721.3
[rd1(-/-)][-][d15] [Notreatment][retina][mm]
173.6
629.4
[rd1(-/-)][-][d35] [Notreatment][retina][mm]
136.4
94.3

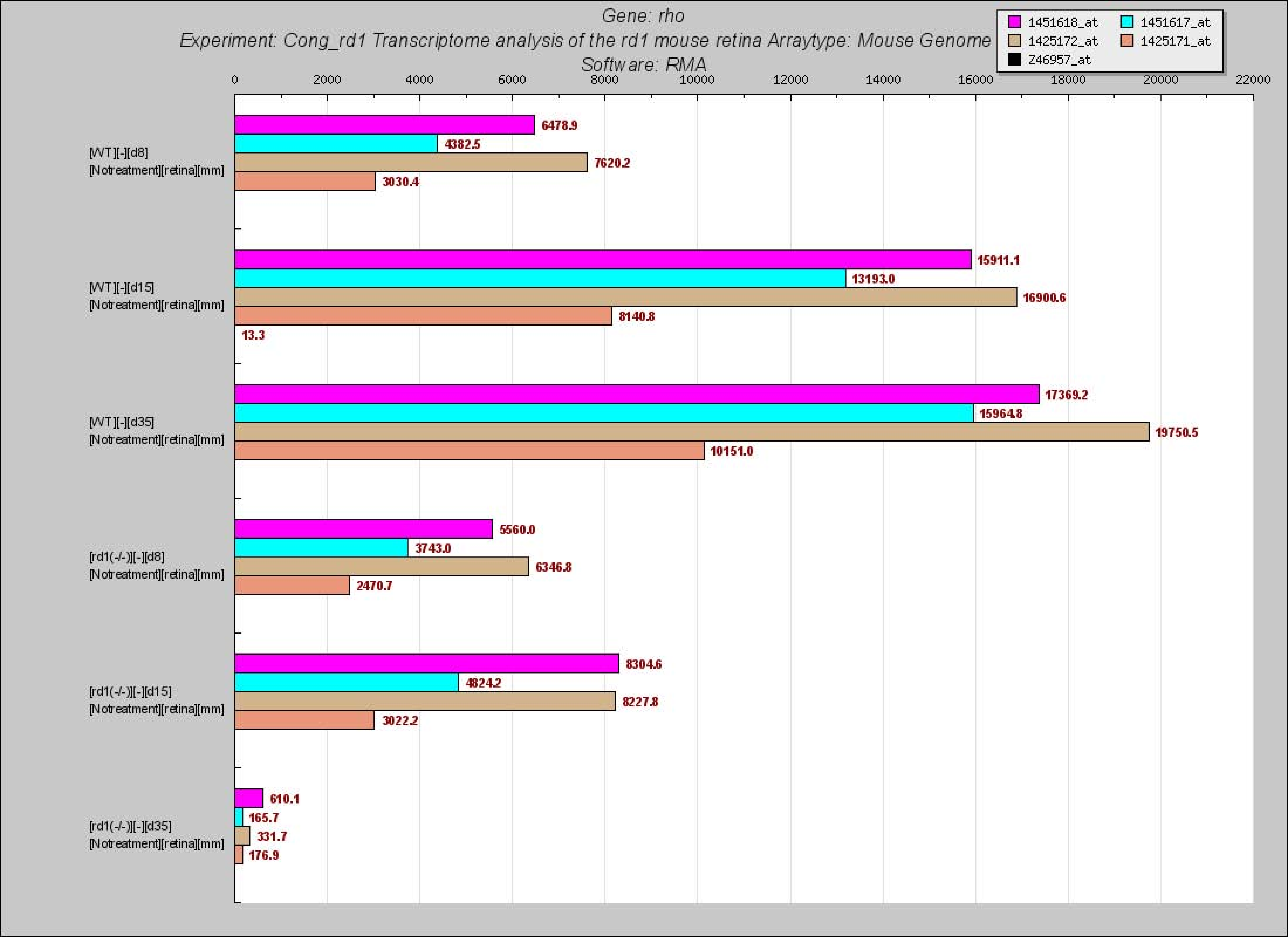

Gene: rho
Experiment: Cong_rd1 Transcriptome analysis of the rd1 mouse retina Arraytype: Mouse Genome
Software: RMA
1451618_at
1451617_at
1425172_at
1425171_at
Z46957_at
0
2000
4000
6000
8000
10000
12000
14000
16000
18000
20000
22000
[WT][-][d8] [Notreatment][retina][mm]
6478.9
4382.5
7620.2
3030.4
[WT][-][d15] [Notreatment][retina][mm]
15911.1
13193.0
16900.6
8140.8
13.3
[WT][-][d35] [Notreatment][retina][mm]
17369.2
15964.8
19750.5
10151.0
[rd1(-/-)][-][d8] [Notreatment][retina][mm]
5560.0
3743.0
6346.8
2470.7
[rd1(-/-)][-][d15] [Notreatment][retina][mm]
8304.6
4824.2
8227.8
3022.2
[rd1(-/-)][-][d35] [Notreatment][retina][mm]
610.1
165.7
331.7
176.9
