# Supplementary material for: Rod-derived Cone Viability Factor-2 is a novel bifunctional-thioredoxin-like protein with therapeutic potential
Source: BMC Mol Biol. 2007 Aug 31;8:74. doi: 10.1186/1471-2199-8-74 (PMC2064930; doi:10.1186/1471-2199-8-74)
Supplement: Additional file 4 — Tissue distribution of RdCVF and RdCVF2 mRNAs. The table describes tissue expression of the EST and mRNA sequences available in the EMBL databases corresponding to RdCVF(-L/-S/2-L/2-S) transcripts. [file 1471-2199-8-74-S4.ppt]

## Slide 1
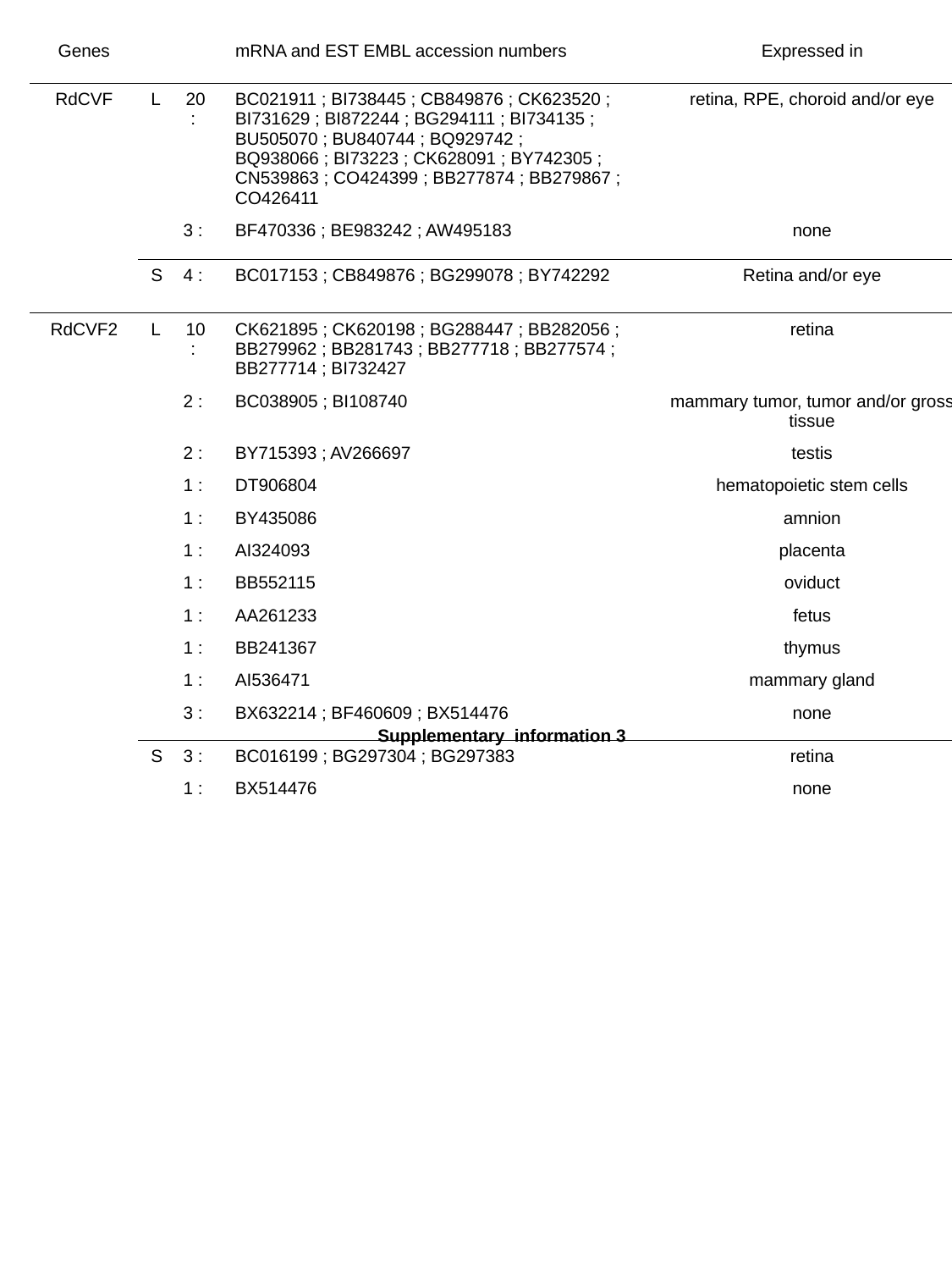

| Genes | | mRNA and EST EMBL accession numbers | | Expressed in |
| --- | --- | --- | --- | --- |
| RdCVF | L | 20 : | BC021911 ; BI738445 ; CB849876 ; CK623520 ; BI731629 ; BI872244 ; BG294111 ; BI734135 ; BU505070 ; BU840744 ; BQ929742 ; BQ938066 ; BI73223 ; CK628091 ; BY742305 ; CN539863 ; CO424399 ; BB277874 ; BB279867 ; CO426411 | retina, RPE, choroid and/or eye |
| | | 3 : | BF470336 ; BE983242 ; AW495183 | none |
| | S | 4 : | BC017153 ; CB849876 ; BG299078 ; BY742292 | Retina and/or eye |
| RdCVF2 | L | 10 : | CK621895 ; CK620198 ; BG288447 ; BB282056 ; BB279962 ; BB281743 ; BB277718 ; BB277574 ; BB277714 ; BI732427 | retina |
| | | 2 : | BC038905 ; BI108740 | mammary tumor, tumor and/or gross tissue |
| | | 2 : | BY715393 ; AV266697 | testis |
| | | 1 : | DT906804 | hematopoietic stem cells |
| | | 1 : | BY435086 | amnion |
| | | 1 : | AI324093 | placenta |
| | | 1 : | BB552115 | oviduct |
| | | 1 : | AA261233 | fetus |
| | | 1 : | BB241367 | thymus |
| | | 1 : | AI536471 | mammary gland |
| | | 3 : | BX632214 ; BF460609 ; BX514476 | none |
| | S | 3 : | BC016199 ; BG297304 ; BG297383 | retina |
| | | 1 : | BX514476 | none |
Supplementary information 3
